# Co–In Bimetallic Hydroxide Nanosheet Arrays With Coexisting Hydroxyl and Metal Vacancies Anchored on Rod‐Like MOF Template for Enhanced Photocatalytic CO_2_ Reduction

**DOI:** 10.1002/advs.202411673

**Published:** 2024-12-04

**Authors:** Jingjuan Feng, Weiwei Li, Tianxia Chen, Zhaopeng Zeng, Meng Tian, Wenxin Ji, Yan Guo, Shixiong Min, Xiangyu Liu

**Affiliations:** ^1^ State Key Laboratory of High‐efficiency Utilization of Coal and Green Chemical Engineering College of Chemistry and Chemical Engineering Ningxia University Yinchuan 750021 China; ^2^ School of Chemistry and Chemical Engineering North Minzu University Yinchuan 750021 China

**Keywords:** catalytic reduction, CO_2_ photoreduction, layered double hydroxides, metal‐organic framework, ultrathin nanosheets

## Abstract

Layered double hydroxides (LDHs) can serves as catalysts for CO_2_ photocatalytic reduction (CO_2_PR). However, the conventionally synthesized LDHs undergo undesired aggregation, which results in an insufficient number of active sites and limits the desirable electron transfer required for CO_2_PR. The metal‐organic framework (MOF) template‐grown LDHs demonstrate excellent promise for exploiting the strengths of both MOFs and LDHs. Herein, the in situ growth of MIL‐68(In)‐NH_2_ MOF‐templated Co–In bimetallic catalyst (CoIn‐LDH/MOF) having an ultrathin nanosheet morphology on the preserved rod‐like MOF template is demonstrated. Compared to the conventionally grown bimetallic LDH (CoIn‐LDH), CoIn‐LDH/MOF not only exposes more active sites but also possesses hydroxyl vacancies (*V*_OH_) and Co vacancies (*V*_Co_). Thus, CoIn‐LDH/MOF performs a higher CO generation rate of 2320 µmol g^−1^ h^−1^ during CO_2_PR, demonstrating improved activity and selectivity than those in CoIn‐LDH. Experiments coupled with calculations reveal that the CoIn‐LDH/MOF‐driven CO_2_PR follows the ^*^COOH pathway. The lower energy barriers for the formation of ^*^COOH and CO(g) can be attributed to the coexistence of *V*_OH_ and *V*_Co_ in CoIn‐LDH/MOF, effectively promoting charge transfer and enhancing CO_2_PR performance. This study provides a new strategy to obtain high‐performant LDH‐based catalysts with improved morphology.

## Graphical abstract

A newly prepared and unreported In/Co bimetallic LDH catalyst is developed, which is in‐situ grown on a rod‐like MOF template and characteristic of ultrathin nanosheet morphology. The resulting catalyst with simultaneous attendance of hydroxyl vacancies and metal vacancies exhibits an enhanced activity of photocatalytic CO_2_ to CO, as compared to pristine MOF and conventionally prepared CoIn‐LDH.

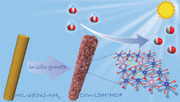

## 1 Introduction

Solar light‐induced conversion of CO_2_ into valuable products, such as CO, CH_4_, or HCOOH, is a useful technique for mitigating CO_2_ emissions and making the recycling of CO_2_ economically viable.^[^ ^1^, ^2^ ^]^ Particularly, CO is an important C1 chemical for the production of other chemicals, such as alcohols, ethers, acids, esters, and their derivatives.^[^ ^3^ ^]^ However, the existing technologies for the CO_2_ photocatalytic reduction (CO_2_PR) process are far from commercialization because of inadequate light absorption, fast recombination of electrons and holes, and ultrahigh C═O dissociation energy (≈750 kJ mol^−1^). The inefficient charge separation and limited availability of active sites further contribute to the low photocatalytic activity of the existing catalysts.^[^ ^4^ ^]^ Additionally, the reduction of H_2_O to H_2_ and photogenerated electrons interferes with CO_2_PR, resulting in low selectivity for C products. Therefore, the development of catalysts that can efficiently promote the photoreduction of CO_2_ under visible light (*λ* > 400 nm) is highly desirable for the solar‐to‐fuel conversion.^[^ ^5^ ^]^

Layered double hydroxides (LDHs) are an important class of 2D materials with tunable chemical components and structural characteristics.^[^ ^6^ ^]^ Recently, various LDHs have been explored as catalysts for the CO_2_PR process. Typically, LDHs have a high capacity for CO_2_ adsorption, large surface area, and highly exposed active sites. However, the conventionally synthesized LDHs often aggregate to form bulky motifs with small surface areas and low light‐absorption capacities, hindering the CO_2_PR process.^[^ ^7^, ^8^ ^]^ Therefore, the development of a new synthetic strategy to obtain highly active LDH‐based catalysts with improved morphology is essential.

Metal‐organic frameworks (MOFs), composed of organic ligands with metal ions, represent high‐porosity crystalline materials.^[^ ^9^ ^]^ In MOFs, electrons can interactively transfer from metal cations to ligands, making them suitable for use in the CO_2_PR process. However, in most cases, the relatively wide bandgaps of MOFs result in lower solar energy absorption and limit the direct use of MOFs in the CO_2_PR process. Moreover, the low electron transfer rate and quantum efficiency of MOFs also hinder their photocatalytic activity.^[^ ^10^ ^]^ Previous studies have established that MOFs can be transformed into functional LDHs or utilized as substrates to grow structurally dispersed LDHs with specific morphology, avoiding the undesirable aggregation of LDHs that results from the conventional synthetic route.^[^ ^8^ ^]^ MOF‐templated LDHs promote the formation of ultrathin 2D nanosheets with multiple active sites and structures, such as vacancies, which can modulate the band gap and electronic structure of catalysts, facilitating the separation of photogenerated electron‐hole pairs.^[^ ^6^ ^]^

Indium (III)‐based materials are considered ideal catalysts because 5s5p orbitals can undergo hybridization with O 2p or S 3p orbitals in oxides or sulfides, respectively, leading to the formation of dispersive band gaps and narrow bands for visible‐light absorption. Furthermore, the physicochemical compatibility of In^3+^ atoms with most metals allows for the tunable surface atomic structure of In‐based materials.^[^ ^11^ ^]^ In this study, a conventionally grown bimetallic Co–In LDH‐based nanosheet (CoIn‐LDH) is compared with an in situ grown MIL‐68(In)‐NH_2_ MOF‐templated CoIn‐LDH/MOF‐based nanosheet having a rod‐like morphology. The synthesized CoIn‐LDH composite exhibits an aggregated morphology with unexposed active sites and only hydroxyl vacancies (*V*_OH_), whereas the well‐designed CoIn‐LDH/MOF composite exhibits abundant active sites provided by their unique lamellar architecture and the coexistence of *V*_OH_ and Co vacancies (*V*_Co_). The presence of vacancies modifies the surrounding environment of metal centers to modulate the charge density and increase the electron concentration.^[^ ^12^ ^]^ Thus, CoIn‐LDH/MOF results in a higher CO generation rate of 2320 µmol g^−1^ h^−1^ following CO_2_ reduction under visible light (*λ* > 400 nm), outperforming CoIn‐LDH alone. A combined analysis of experimental and theoretical results indicates that the energy barriers for the formation of ^*^COOH and CO(g) during the photocatalytic CO_2_ reduction performed using CoIn‐LDH/MOF as the catalyst are lower than those obtained using CoIn‐LDH. The results of this study highlight that the presence of *V*_OH_ and *V*_Co_ in CoIn‐LDH/MOF provides an effective route for the charge to transfer, enhancing the CO_2_PR performance.

## 2 Results and Discussion

### 2.1 Synthesis and Characterization

**Scheme 1:**
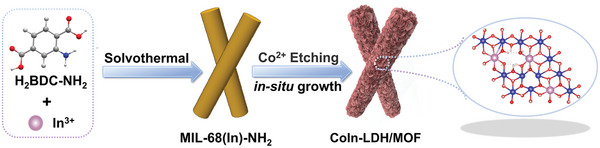
Schematic for the synthetic process of CoIn‐LDH/MOF.

The synthetic of CoIn‐LDH/MOF is illustrated in **Scheme** **1**. The solvothermal method was used for synthesizing MOF MIL‐68(In)‐NH_2_ according to a previous report.^[^ ^13^ ^]^ Subsequently, the synthesized MOF MIL‐68(In)‐NH_2_ was etched using energetic Co^2+^ ions and hydrolyzed In^3+^ ions to form CoIn‐LDH/MOF via co‐precipitation (details are provided in the supplementary information). The transformation of MOF into LDH was confirmed by comparing the etching rate of the MOF and the generation rate of the LDH. The reaction conditions, such as the concentration of Co^2+^ ions, etching time, and solvent were adjusted to obtain CoIn‐LDH/MOF with desired structural properties and behavior. Particularly, the solvent composition was adjusted by mixing water and ethanol in a 3:1 ratio to promote metal ion hydrolysis, which is limited in anhydrous ethanol but can be improved by adding water. Urea and ammonium fluoride were added to the experimental solution to adjust the pH and accelerate the formation of CoIn‐LDH/MOF, as well as to support the interlayer anions.^[^ ^8^, ^14^ ^]^

**Figure 1:**
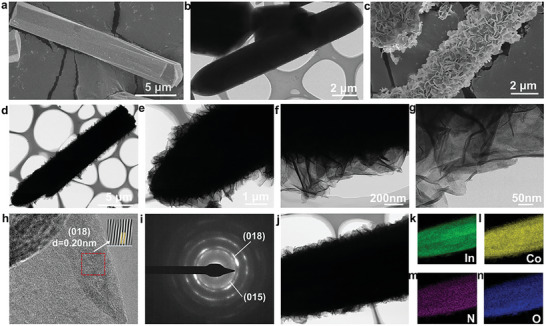
a) SEM and b) TEM images of MIL‐68(In)‐NH_2_. c) SEM images, d–g) TEM images, h) HRTEM images, i) SAED patterns, and j–n) elemental mapping of CoIn‐LDH/MOF.

Scanning electron microscopy (SEM) and transmission electron microscopy (TEM) were used to examine the morphology of the as‐prepared materials. The MOF MIL‐68(In)‐NH_2_ has a solid rod‐like structure with a smooth surface (**Figure** **1**a,b), whereas the CoIn‐LDH/MOF has an ultrathin nanosheet morphology and retains the rod‐like structure of the MOF template (Figure 1c and Figure S1, Supporting Information). The morphology of CoIn‐LDH/MOF is clearly visible in the TEM images recorded at different magnifications (Figure 1d–g). It can be speculated that In^3+^ ions detach from the MOF structure to precipitate externally; thus, conversions appear on the surface of the MOF, forming the shell of the LDH. During Co^2+^ ion etching, hierarchical pores are established on the synthesized CoIn‐LDH/MOF, promoting mass diffusion and providing fascinating surface chemistry.^[^ ^14^ ^]^ SEM observations revealed that CoIn‐LDH has a bulky morphology, which is possibly caused by the aggregation of CoIn‐LDH nanosheets during the synthesis process (Figure S2, Supporting Information). High‐resolution TEM (HRTEM) images of CoIn‐LDH/MOF show a lattice spacing of 0.20 nm attributed to the (018) plane of CoIn‐LDH/MOF (Figure 1h). The two diffraction rings, attributed to the (018) and (015) planes of CoIn‐LDH/MOF, in the selected area electron diffraction (SAED) pattern (Figure 1i) confirm the formation of CoIn‐LDH/MOF. In addition, elemental mapping indicates that In, Co, O, N, and C are uniformly distributed across the rod‐like structures of CoIn‐LDH/MOF (Figure 1j–n). Additionally, the elemental maps corresponding to C and N indicate that the organic ligands are incompletely etched (Figure S3, Supporting Information).

The powder X‐ray diffraction (PXRD) pattern of the synthesized MIL‐68(In)‐NH_2_ is consistent with the simulated XRD pattern (Figure S4, Supporting Information). In the PXRD patterns of CoIn‐LDH/MOF and CoIn‐LDH, the presence of signals corresponding to the (003), (006), (009), (015), (018), (110), and (113) planes at 2*θ* values of 11.36°, 22.45°, 33.37°, 37.89°, 45.31°, 57.98°, and 59.17°, respectively, indicate a well‐crystallized hexagonal layered morphology. As shown in Figure S4 (Supporting Information), CoIn‐LDH/MOF retains a portion of structures of MIL‐68(In)‐NH_2_.^[^ ^8b^ ^]^ Fourier‐transform infrared (FTIR) spectroscopy was used to characterize the chemical structures of MIL‐68(In)‐NH_2_, CoIn‐LDH/MOF, and CoIn‐LDH (Figure S5, Supporting Information). The FTIR spectrum of MIL‐68(In)‐NH_2_ matches well with that presented in a previous report.^[^ ^15^ ^]^ The broad bands at 3438 and 3395 cm^−1^ in the FTIR spectra of CoIn‐LDH/MOF and CoIn‐LDH, respectively, correspond to the O─H stretching vibrations of ─OH units in the LDH nanosheets and interlayer H_2_O molecules. The peaks at 1572 and 1648 cm^−1^ correspond to the bending vibrations of the interlayer H_2_O molecules in CoIn‐LDH/MOF and CoIn‐LDH, respectively.^[^ ^16^ ^]^ The intense bands at 1364 and 1352 cm^−1^ in the FTIR spectra of CoIn‐LDH/MOF and CoIn‐LDH, respectively, are associated with the vibrations of the CO_3_^2−^ anions. The low‐intensity vibrational bands that are present in the FTIR spectrum of MIL‐68(In)‐NH_2_ are also present in the FTIR spectrum of CoIn‐LDH/MOF, indicating that the partial structure of the MOF MIL‐68(In)‐NH_2_ template is retained in the structure of CoIn‐LDH/MOF. These results are consistent with the elemental mapping experiment and confirm the successful synthesis of MIL‐68(In)‐NH_2_, CoIn‐LDH/MOF, and CoIn‐LDH.

**Figure 2:**
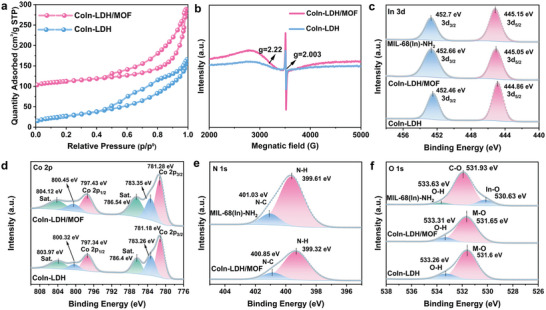
a) N_2_ adsorption/desorption isotherms of CoIn‐LDH/MOF and CoIn‐LDH. b) EPR spectra of CoIn‐LDH/MOF and CoIn‐LDH. c) In 3d core‐level XPS spectra of MIL‐68(In)‐NH_2_, CoIn‐LDH/MOF, and CoIn‐LDH. d) Co 2p core‐level XPS spectra of CoIn‐LDH/MOF, and CoIn‐LDH. e) N 1s core‐level XPS spectra MIL‐68(In)‐NH_2_ and CoIn‐LDH/MOF. f) O 1s core‐level XPS spectra of MIL‐68(In)‐NH_2_, CoIn‐LDH/MOF, and CoIn‐LDH.

The Brunauer–Emmett–Teller (BET) measurements show that CoIn‐LDH/MOF exhibits a larger BET surface area (*S*_BET_ = 102 m^2^ g^−1^) than that of CoIn‐LDH (*S*_BET_ = 66 m^2^ g^−1^) (**Figure** **2**a), indicating that CoIn‐LDH/MOF has more active sites for accelerating surface reaction rates and improving the mass transfer of CO_2_ and H_2_O, both of which are favorable for CO_2_PR in a liquid–solid system.^[^ ^17^, ^18^ ^]^ The electron paramagnetic resonance (EPR) measurement was utilized to record information about the unpaired electrons. For MIL‐68(In)‐NH_2_, there is no obvious signal in the EPR spectrum (Figure S6, Supporting Information), suggesting no defects in MIL‐68(In)‐NH_2_. However, an intense signal at g = 2.003 indicates the presence of hydroxyl vacancies (*V*_OH_) in both CoIn‐LDH and CoIn‐LDH/MOF (Figure 2b), and the *V*_OH_ concentration of CoIn‐LDH/MOF is higher, implying the increase of the number of unpaired electrons on the surface of the metal sites, and thus facilitating CO_2_PR. Compared with CoIn‐LDH, CoIn‐LDH/MOF shows a more prominent EPR signal at *g* = 2.22 related to Co vacancies (*V*_Co_) which can modulate the redistribution of catalyst electrons.^[^ ^19^ ^]^ Inductively coupled plasma experiments were conducted to study the elemental compositions of the CoIn‐LDH/MOF composites, with an In/Co ratio of ≈1:3 (Table S1, Supporting Information).

X‐ray photoelectron spectroscopy (XPS) was used to probe the electronic and chemical structures of MIL‐68(In)‐NH_2_, CoIn‐LDH/MOF, and CoIn‐LDH. The core‐level XPS spectra of In, O, C, and N of MIL‐68(In)‐NH_2_, In, Co, O, N, and C of CoIn‐LDH/MOF, and In, Co, and O of CoIn‐LDH are shown in Figure S7 (Supporting Information). As shown in Figure 2c, the In 3d core‐level XPS spectra of MIL‐68(In)‐NH_2_, CoIn‐LDH/MOF, and CoIn‐LDH show peaks at 445.15, 445.05, and 444.86 eV, respectively, corresponding to In 3d_5/2_, and 452.70, 452.66, and 452.46 eV, respectively, corresponding to In 3d_3/2_, indicating the presence of In^3+^ cation.^[^ ^13^ ^]^ The In 3d core‐level spectra of CoIn‐LDH/MOF are shifted toward lower binding energies compared to that of MIL‐68(In)‐NH_2_, indicating a different electronic structure for In and extent of electron transfer among In, O, and Co species in CoIn‐LDH/MOF. Notably, the Co 2p core‐level spectra (Figure 2d) of CoIn‐LDH/MOF and CoIn‐LDH show two peaks corresponding to Co^2+^ and Co^3+^ ions, as well as two satellite peaks corresponding to Co^2+^ ions. Because the raw materials used for synthesizing LDHs involve only Co^2+^ ions, the presence of Co^3+^ ions in the composites is possibly due to the partial oxidation of Co^2+^ resulting from the Jahn–Teller effect.^[^ ^19^ ^]^ In the Co 2p core‐level spectra of CoIn‐LDH/MOF, the peaks at 781.28 and 783.35 eV correspond to Co^3+^ and Co^2+^, respectively. In the Co 2p core‐level spectra of CoIn‐LDH, the peaks at 781.18 and 783.26 eV correspond to Co^3+^ and Co^2+^, respectively. In the N 1s core‐level spectra (Figure 2e), the peaks at 399.61 and 401.03 eV in MIL‐68(In)‐NH_2_ and 399.32 and 400.85 eV in CoIn‐ LDH/ MOF can be assigned to N─H and N─C bonds, respectively, confirming that the partial structure of the MOF MIL‐68(In)‐NH_2_ template is retained structure of CoIn‐LDH/MOF.^[^ ^15^ ^]^ The O1s core‐level spectrum of MIL‐68(In)‐NH_2_ shows three peaks at 530.63, 531.93, and 533.63 eV corresponding to In─O, C─O, and O─H bonds, respectively (Figure 2f). The O─H moieties represent the H_2_O molecules on the surface of MIL‐68(In)‐NH_2_. Fitting the O 1s core‐level spectrum of CoIn‐LDH/MOF reveals the presence of two peaks at 531.65 and 533.31 eV corresponding to M─O and O─H bonds, respectively. The two deconvoluted peaks at 531.60 and 533.26 eV in the O 1s core‐level spectrum of CoIn‐LDH can be attributed to M─O and O─H bonds, respectively.^[^ ^20^ ^]^

**Figure 3:**
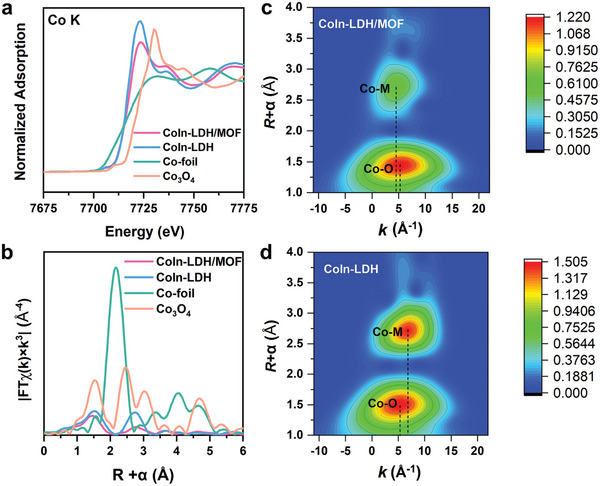
a) Normalized XANES spectra Co K‐edge. b) FT‐EXAFS spectra Co K‐edge. WT‐EXAFS spectra of Co K‐edge for c) CoIn‐LDH/MOF and d) CoIn‐LDH.

The X‐ray absorption near edge structure (XANES) and extended X‐ray adsorption fine structure (EXAFS) were further used to investigate the structure of as‐synthesized CoIn‐LDH/MOF and CoIn‐LDH. A similar absorption edge position to each other of Co Kedge XANES spectra exhibits that the valences of Co atoms are similar between CoIn‐LDH/MOF and CoIn‐LDH (**Figure** **3**a). Fourier‐transformed (FT) k^3^‐weighted EXAFS spectra (Figure 3b) display two peaks centered at 1.97 and 2.95 Å for CoIn‐LDH/MOF, and 2.02 and 3.14 Å for CoIn‐LDH, respectively, which could be assigned to the Co‐O coordination shell and Co‐O‐M coordination shell. Besides, the results of the EXAFS data fitting analysis are summarized in Table S2 (Supporting Information). The average coordination numbers (CNs) of Co‐O (3.7±0.4) or Co‐O‐M (4.6±1.0) in CoIn‐LDH/MOF are smaller than Co‐O (6.4±0.6) or Co‐O‐M (5.7±0.7) in CoIn‐LDH, which is attributed to the increased hydroxyl vacancies and metal vacancies during the structurally topological transformation. The wavelet transform plots also visually demonstrate the presence of two shell layers (Co─O and Cu─O─M) in CoIn‐LDH/MOF and CoIn‐LDH (Figure 3c,d). The *k* value of Co‐M is slightly smaller than Co‐O for CoIn‐LDH/MOF due to the impact of other scattering paths.

### 2.2 Photochemical CO_2_ Reduction Performance

For a thorough comparison, CO_2_PR was attempted using all the synthesized materials in a solution of acetonitrile in H_2_O under visible‐light irradiation. [Ru(bpy)_3_]Cl_2_ and triethanolamine (TEOA) were selected as the photosensitizer and sacrificial agent, respectively. ^1^H NMR characterization indicated that no liquid products, such as HCHO, HCOOH, or CH_3_OH, were formed during CO_2_PR performed using CoIn‐LDH/MOF (Figure S8, Supporting Information). Gas chromatography was used to detect and analyze the gaseous products of the CO_2_PR reaction.

**Figure 4:**
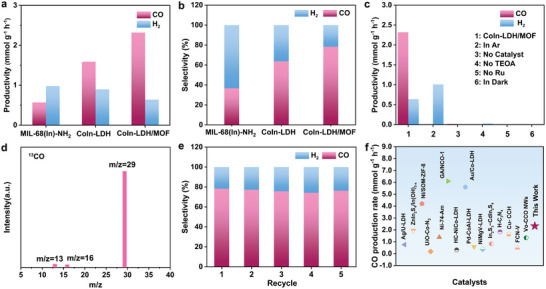
a) Production rate and b) selectivity toward CO and H_2_ of the catalysts. c) Rates of CO and H_2_ evolution during CO_2_PR under different reaction conditions. d) Mass spectrum of photocatalytic products generated during isotopic ^13^CO_2_ labeled photoreduction experiment. e) Results of the cycling stability tests determining selectivity toward CO and H_2_, using CoIn‐LDH/MOF as the catalyst. f) Comparison of the CO production rates obtained from different catalysts under the same conditions (In_2_S_3_‐CdIn_2_S_3_ in 2017, others catalysts in 2020–2024).

As depicted in **Figure** **4**a, the performances of MIL‐68(In)‐NH_2_, CoIn‐LDH/MOF, and CoIn‐LDH as catalysts are significantly different. The products of the catalytic process are CO and H_2_. The determined CO yields are 2.32, 0.6, and 1.58 mmol g^−1^ h^−1^ using CoIn‐LDH/MOF, MIL‐68(In)‐NH_2_, and CoIn‐LDH, respectively. The determined H_2_ yields are 0.64, 0.98, and 0.90 mmol g^−1^ h^−1^ using CoIn‐LDH/MOF, MIL‐68(In)‐NH_2_, and CoIn‐LDH, respectively. Among the three catalysts, CoIn‐LDH/MOF exhibits the highest CO yield, demonstrating significantly enhanced CO selectivity (Figure 4b). MIL‐68(In)‐NH_2_ is almost inactive toward CO_2_PR. When the catalytic activity is normalized by the surface area, CoIn‐LDH/MOF and CoIn‐LDH present an approximate CO yield but significantly different H_2_ yields (Table S3, Supporting Information), supporting that CoIn‐LDH/MOF possesses a higher selectivity due to more site exposure. Compared with CoIn‐LDH, CoIn‐LDH/MOF exhibits more uniformly exposed active sites in the hierarchically porous structure and optimized electronic structure owing to the presence of *V*_OH_ and *V*_Co_, facilitating separation and improved mobility of the charge carriers.

To identify the factors affecting the CO_2_PR using CoIn‐LDH/MOF, control experiments were performed under various reaction conditions (Figure 4c). Relatively small amounts of H_2_ and no CO were produced when Ar was replaced with CO_2_ during the photocatalytic reaction, indicating that CO production originates from CO_2_. The removal of TEOA from the system significantly reduced the catalytic activity because TEOA depleted the photogenerated holes and decreased the recombination rate of photogenerated carriers. Similarly, no products were observed in the absence of the Ru‐based photosensitizer, indicating that the photogenerated electrons are generated as a consequence of Ru photosensitization. The removal of the catalyst or light source generated no products, confirming that the CoIn‐LDH/MOF catalyst with active sites for CO_2_ reduction is essential and that the catalytic process is driven by light.

To determine the carbon sources of the C‐containing products of the photoreduction process, isotope labeling experiments were conducted using isotopic ^13^CO_2_ as the reactant. The mass spectrum of the CO species was determined using a ^13^CO_2_ substrate under the same reaction conditions. As shown in Figure 4d, the peak at *m*/*z* = 29, corresponding to ^13^CO, demonstrates that the CO species are generated from CO_2_PR. Experiments on the durability and continuous cycling stability of the catalysts are essential for practical applications. As shown in Figure 4e, the results of the cycling stability tests show that CoIn‐LDH/MOF catalyst has excellent catalytic activity, with a CO selectivity of ≈78% after recycling for at least five cycles. After the catalytic reaction, the element distribution of CoIn‐LDH/MOF materials remains unchanged, as confirmed by TEM and elemental mapping (Figure S9, Supporting Information). For comparison, the CO production rates of the present and reported catalysts are presented in Figure 4f, indicating that CoIn‐LDH/MOF exhibits an efficient CO_2_PR performance.^[^ ^1^, ^12^, ^16^, ^20^, ^21^, ^22^, ^23^, ^24^, ^25^, ^26^, ^27^, ^28^, ^29^, ^30^, ^31^ ^]^

### 2.3 Photoelectric Characterization

**Figure 5:**
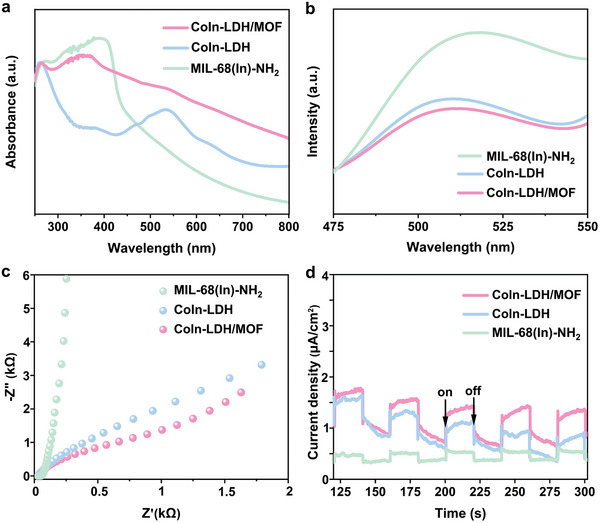
a) UV–vis spectra, b) photoluminescence spectra under 360 nm excitation, c) electrochemical impedance spectra, and d) photocurrent–time profiles of MIL‐68(In)‐NH_2_, CoIn‐LDH/MOF, and CoIn‐LDH.

The photoelectric properties of the catalysts were investigated to identify the separation and transfer of charge carriers. Although all three catalysts can absorb ultraviolet (UV) and visible light in the 250–800 nm range (**Figure** **5**a), CoIn‐LDH/MOF can absorb relatively more amount of light. The photoluminescence experiments of MIL‐68(In)‐NH_2_, CoIn‐LDH/MOF, and CoIn‐LDH were carried out under 360 nm excitation.^[^ ^1c,d^ ^]^ As compared to MIL‐68(In)‐NH_2_ and CoIn‐LDH, CoIn‐LDH/MOF exhibit the lowest photoluminescence (PL) intensity (Figure 5b) under the same excitation, indicating the most effective inhibition of carrier recombination. The electrochemical impedance spectra of MIL‐68(In)‐NH_2_, CoIn‐LDH, and CoIn‐LDH/MOF are also compared and analyzed. Among all the three catalysts, CoIn‐LDH/MOF exhibits the smallest arc radius in the electrochemical impedance spectrum (Figure 5c), indicating that the CoIn‐LDH/MOF catalyst is the most effective in interfacial charge transfer. According to the results of electrochemical impedance and PL spectroscopies, the formation of a hierarchical structure effectively separates carriers by providing a shorter carrier migration distance, hence reducing carrier recombination during the transport process. Another possible reason for the reduction of carrier recombination is the formation of *V*_OH_ and *V*_Co_. As depicted in Figure 5d, the photocurrent density of CoIn‐LDH/MOF is higher than those of MIL‐68(In)‐NH_2_ and CoIn‐LDH, indicating a higher electron yield in the reduction half‐reaction. Thus, the generation, separation, and transfer of photoinduced charges are effectively facilitated by the CoIn‐LDH/MOF catalyst, resulting in highly efficient CO_2_PR activity.

### 2.4 Mechanism of CO_2_ Photoreduction

**Figure 6:**
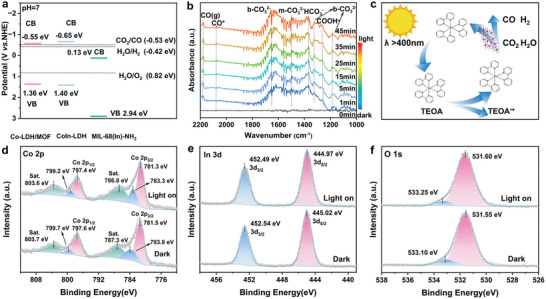
a) Band edge positions of CoIn‐LDH/MOF, CoIn‐LDH, and MIL‐68(In)‐NH_2_. b) In situ, DRIFT spectra of CoIn‐LDH/MOF recorded during CO_2_ reduction. c) Plausible reaction process for the photocatalytic CO_2_ reduction using CoIn‐LDH/MOF. d) Co 2p, e) In 3d, and f) O 1s core‐level spectra, recorded in situ, of irradiated CoIn‐LDH/MOF.

XPS valence band (VB) spectra and Tauc curves were obtained to study the band positions of the MIL‐68(In)‐NH_2_, CoIn‐LDH/MOF, and CoIn‐LDH catalysts. The VB positions in MIL‐68(In)‐NH_2_, CoIn‐LDH/MOF, and CoIn‐LDH are determined to be 2.94, 1.36, and 1.40 eV, respectively (Figure S10, Supporting Information). Tauc curves were derived using UV–vis diffuse reflectance spectra to extract the bandgap energy (*E*_g_) using the equation *α*(*hν*) = *A*(*hν*‐*E*_g_)^n/2^. The *E*_g_ values of MIL‐68(In)‐NH_2_, CoIn‐LDH, and CoIn‐LDH/MOF are determined to be 2.81, 2.05, and 1.91 eV, respectively (Figure S11, Supporting Information). The lower *E*_g_ of CoIn‐LDH/MOF results in a narrower intrinsic bandgap owing to the coexistence of *V*_OH_ and *V*_Co_, indicating that CoIn‐LDH/MOF possesses superior light‐absorption capacity than MIL‐68(In)‐NH_2_ and CoIn‐LDH to form more photogenerated electrons. The obtained band edge positions of the catalysts are depicted in **Figure** **6**a. The conduction band minimum (CBM) values, estimated using the formula *E*_CB_ = *E*_VB_−*E*_g_, are 0.13, −0.55, and −0.65 eV (vs NHE) for MIL‐68(In)‐NH_2_, CoIn‐LDH/MOF, and CoIn‐LDH, respectively. For MIL‐68(In)‐NH_2_, the conduction band (0.13 V) forbids the proton reduction process. The occurrence of low CO_2_PR activity is resulted from the fact that the cationic photosensitizer, [Ru(bpy)_3_]^2+^, might be absorbed by MIL‐68(In)‐NH_2_ during the stirring process. The estimated CBM values for CoIn‐LDH/MOF and CoIn‐LDH are more negative than the potential for converting CO_2_ to CO (CO_2_/CO: −0.53 V vs NHE at pH = 7), thermodynamically satisfying the photocatalytic reduction of CO_2_ to CO.^[^ ^32^ ^]^

To better define the CO_2_PR process, in situ diffuse reflectance infrared Fourier transform spectroscopy (DRIFTS) measurements were performed for CoIn‐LDH/MOF, and the results are shown in Figure 6b. Under dark conditions, the background signal was recorded after introducing CO_2_ into the reaction. The absence of a signal at 0 min indicates that the CO_2_PR process did not occur. As illumination time increases, peaks are observed at 1657, 1325, and 1184 cm^−1^, indicating the production of bidentate carbonate (b‐CO_3_^2−^).^[^ ^30^, ^33^, ^34^ ^]^ In addition, monodentate carbonate (m‐CO_3_^2−^ at 1500 cm^−1^) is also present, suggesting chemisorption of CO_2_ on the catalyst.^[^ ^17^ ^]^ The peak at 1368 cm^−1^ can be attributed to the vibration absorption of bicarbonate (HCO_3_^−^) that is formed by the reaction of CO_2_ with hydroxyls derived from the dissociation of H_2_O on catalysts.^[^ ^35^ ^]^ The peak at 1265 cm^−1^ corresponding to ^*^COOH reveals that the CO_2_PR process possibly follows the ^*^COOH pathway.^[^ ^36^ ^]^ Additionally, the intensity of the bridging ^*^CO peak at 2070 cm^−1^ increases with the increasing light irradiation time, indicating the generation of CO intermediates (^*^CO) on the photocatalytic surfaces. The peak at 2179 cm^−1^ can be assigned to CO(g).^[^ ^37^ ^]^ Based on the results, a potential mechanism for CO_2_PR can be proposed as:^[^ ^32^, ^38^ ^]^

(1) $$CO_{2}\to^{*}CO_{2}+H^{+}+e^{-}\to^{*}\mathrm{COOH}+H^{+}+e^{-}\to^{*}\mathrm{CO}$$

The intermediate steps are:

(2) $$CO_{2}{+}^{*}\to^{*}CO_{2}$$

(3)

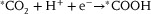



(4)

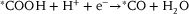



(5)

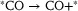




A plausible reaction process is shown in Figure 6c. Initially, the photosensitizer ([Ru(bpy)_3_]^2+^) is excited using visible light, resulting in an excited state species ([Ru(bpy)_3_]^2+*^). The excited photosensitizer ([Ru(bpy)_3_]^2+*^) then degrades to yield reduced [Ru(bpy)_3_]^+^. Subsequently, [Ru(bpy)_3_]^+^ provides excitation electrons to the CoIn‐LDH/MOF catalyst for initiating the CO_2_ reduction reaction. The electrons are transferred to the electrophilic CO_2_ species adsorbed on the surface of the CoIn‐LDH/MOF catalyst, generating ^*^CO intermediates that are desorbed from the coactive sites to produce CO.^[^ ^12^ ^]^

The strong Coulomb attraction between the extranuclear electrons and atomic nucleus is responsible for the electron binding energy of elements. Slight changes in the Coulomb attraction can alter the electron density, indicating that the increase in electron binding energies can be attributed to the decrease in electron density. The electron gaining is usually responsible for the reduction in the electron binding energy.^[^ ^39^ ^]^ The XPS spectrum recorded in situ after irradiation was used to examine the changes in the electron binding energy to determine the direction of charge transfer in CoIn‐LDH/MOF catalyst. As the reaction condition changes from dark to light, the peaks corresponding to Co^3+^ and Co^2+^ ions in the core‐level Co 2p XPS spectra shift toward lower binding energies by 0.2 and 0.5 eV, respectively (Figure 6d); in contrast, the peaks corresponding to In 3d_3/2_ and In 3d_5/2_ ions in the core‐level In 3d XPS spectra shift toward lower binding energies by 0.05 eV (Figure 6e). Contrastingly, the peaks in the core‐level O 2p XPS spectra corresponding to M─O and O─H bonds shift toward higher binding energies by 0.05 and 0.15 eV, respectively, under light irradiation (Figure 6f). It can be concluded that the photogenerated electrons migrate from the O moieties to the In and Co sites under light irradiation, and prefer to transfer to Co sites which can be identified as the active sites during the CO_2_ reduction reaction.^[^ ^40^, ^41^ ^]^

**Figure 7:**
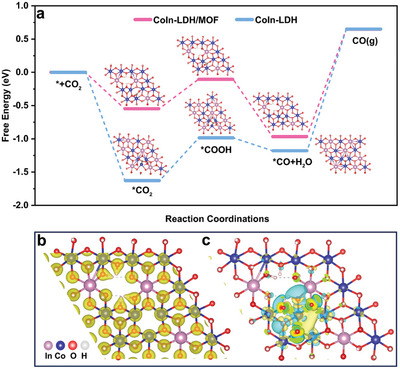
a) Gibbs free energy diagram showing the photocatalytic reduction of CO_2_ to CO using CoIn‐LDH/MOF and CoIn‐LDH. b) Charge density distribution of CoIn‐LDH/MOF (isosurface level: 0.192 eV/Å^3^). c) Differential charge density maps of CoIn‐LDH/MOF (isosurface level: 0.0025 eV Å^−3^) (yellow and blue regions indicate electron accumulation and depletion, respectively).

Finally, density functional theory (DFT) calculations were performed to elucidate the reaction mechanism. Based on the experimental results, the models for CoIn‐LDH/MOF (Co:In = 3:1, *V*_OH_, *V*_Co_), and CoIn‐LDH (Co:In = 1:1, *V*_OH_) were constructed. A model of CoIn‐LDH‐3 (Co:In = 3:1, *V*_OH_) was also joined for comparison (Figures S12–S14, Supporting Information). In three models, the Co atoms were modulated by surface O vacancy to form active sites. In **Figures** **7**a and S15a (Supporting Information), the free energy diagrams showing photocatalytic reduction of CO_2_ to CO using catalysts indicate that ^*^COOH is a crucial intermediate for the production of CO. Further analysis reveals that the rate‐determining step in the CO_2_PR process is the desorption of ^*^CO. The energy barriers for the desorption of ^*^CO are 1.62 eV for CoIn‐LDH/MOF, 1.83 eV for CoIn‐LDH, 1.98 eV for CoIn‐LDH‐3 (Tables S4,S5, Supporting Information). Clearly, the energy barriers for the desorption of ^*^CO on CoIn‐LDH/MOF are the lowest, which is conducive to the CO_2_PR, corresponding to the experimental results (Figure 4a). Therefore, the CoIn‐LDH/MOF advances in thermodynamics and leads to a favorable ^*^CO desorption energy (1.62 eV) for enhancing CO desorption on their surface. The charge density distribution and differential charge density maps were calculated to further elucidate the mechanism. Compared to the CoIn‐LDH (Figure S16a, Supporting Information), the charge distribution in the O atoms around the metal defect is more localized, which makes it easier for electrons to transfer (Figure 7b). The differential charge density (Figure 7c and Figures S15c,S16b, Supporting Information) and Bader charge analysis (Figures S15b,S17 and Tables S6–S8, Supporting Information) synergistically demonstrate that the *V*_Co_ is favorable for electron transfer to lead to a low Gibbs free energy barrier of CO_2_PR.^[^ ^42^ ^]^ Thus, the presence of Co vacancies promotes charge transfer in the CoIn‐LDH/MOF, resulting in a higher electron concentration. Consequently, the CoIn‐LDH/MOF catalyst exhibits a superior CO_2_PR performance.

## 3 Conclusion

A novel hierarchical CoIn‐LDH/MOF was synthesized using an in situ growth strategy starting with MIL‐68(In)‐NH_2_ having a rod‐like morphology. The characterization results showed that the CoIn‐LDH/MOF species adhered to the rod‐like morphology of the MOF template and exhibited an ultrathin nanosheet morphology with a dense hierarchical structure having abundant active sites for CO_2_PR. The CoIn‐LDH/MOF exhibited a higher CO_2_PR activity and CO conversion rate (2.32 mmol g^−1^ h^−1^) compared to those of MIL‐68(In)‐NH_2_ and CoIn‐LDH. The in situ XPS results and DFT calculations revealed that the coexistence of metal and hydroxyl vacancies in CoIn‐LDH/MOF effectively promotes electron transfer, improving the CO_2_PR activity. Further analysis revealed that the CO_2_PR process performed using CoIn‐LDH/MOF as the catalyst involves four conversion steps, during which CoIn‐LDH/MOF effectively desorbs ^*^CO owing to the relatively low formation energy barriers of CO (g). This study highlights the potential of 2D layered materials and provides a promising platform for generating desirable defects that can function as catalytically active sites for solar‐driven photocatalytic CO_2_ conversion.

## Conflict of Interest

The authors declare no conflict of interest.

## Supporting information



Supporting Information

## Data Availability

The data that support the findings of this study are available from the corresponding author upon reasonable request.
