# Supplementary material for: Co–In Bimetallic Hydroxide Nanosheet Arrays With Coexisting Hydroxyl and Metal Vacancies Anchored on Rod‐Like MOF Template for Enhanced Photocatalytic CO2 Reduction
Source: Adv Sci (Weinh). 2024 Dec 4;12(4):2411673. doi: 10.1002/advs.202411673 (PMC11775564; doi:10.1002/advs.202411673)
Supplement: Supplementary file 1 — Supporting Information [file ADVS-12-2411673-s001.docx]

Supporting Information

**Co–In Bimetallic Hydroxide Nanosheet Arrays with Coexisting Hydroxyl and Metal Vacancies Anchored on Rod-like MOF Template for Enhanced Photocatalytic CO_2_ Reduction**

*Jingjuan Feng, Weiwei Li, Tianxia Chen, Zhaopeng Zeng, Meng Tian*, Wenxin Ji, Yan Guo, Shixiong Min, and Xiangyu Liu**

State Key Laboratory of High-efficiency Utilization of Coal and Green Chemical Engineering, College of Chemistry and Chemical Engineering, NingXia University, Yinchuan 750021, China

School of Chemistry and Chemical Engineering, North Minzu University, Yinchuan 750021, China

E-mail: xiangyuliu432@126.com; tianm2023@nxu.edu.cn

**Contents**

**Experimental Section**

**Figure S1.** SEM images of CoIn-LDH/MOF.

**Figure S2.** SEM images of CoIn-LDH.

**Figure S3.** Elemental mapping image of CoIn-LDH/MOF.

**Figure S4.** PXRD patterns for MIL-68(In)-NH_2_, CoIn-LDH/MOF and CoIn-LDH.

**Figure S5.** FT-IR spectra of MIL-68(In)-NH_2_, CoIn-LDH and CoIn-LDH/MOF.

**Figure S6.** EPR spectra of MIL-68(In)-NH_2_.

**Figure S7.** XPS survey spectra of (a) MIL-68(In)-NH_2_, (b)CoIn-LDH/MOF, (c) CoIn-LDH.

**Figure S8.** ^1^H NMR spectra of the reaction system following CO_2_ reduction under illumination (*λ* > 400 nm).

**Figure S9. (**a-b**)** TEM images, (c-h) elemental mapping of CoIn-LDH/MOF after CO_2_ reduction reaction.

**Figure S10.** Valence band XPS spectra of (a) MIL-68(In)-NH_2_, (b)CoIn-LDH/MOF, (c) CoIn-LDH.

**Figure S11.** Tauc plots of (a) MIL-68(In)-NH_2_, (b)CoIn-LDH/MOF, (c) CoIn-LDH.

**Figure S12.** Optimized geometries of photocatalytic CO_2_ reduction intermediates for CoIn LDH/MOF.

**Figure S13.** Optimized geometries of photocatalytic CO_2_ reduction intermediates for CoIn-LDH.

**Figure S14.** Optimized geometries of photocatalytic CO_2_ reduction intermediates for CoIn-LDH-3.

**Figure S15.** (a) Gibbs free energy diagram showing the photocatalytic reduction of CO_2_ to CO using CoIn-LDH-3. (b) Bader charge analysis, (c) Differential charge density maps of CoIn-LDH-3 (isosurface level: 0.0025 eV/Å^3^) (yellow and blue regions indicate electron accumulation and depletion, respectively).

**Figure S16.** (a) Charge density distribution of CoIn-LDH (isosurface level: 0.192 eV/Å^3^). (b) Differential charge density maps of CoIn-LDH (isosurface level: 0.0025 eV/Å^3^) (yellow and blue regions indicate electron accumulation and depletion respectively).

**Figure S17.** Bader charge analysis of (a) CoIn-LDH/MOF, (b) CoIn-LDH.

**Table S1** Summary of elemental analyses data for CoIn-LDH/MOF and CoIn-LDH from ICP.

**Table S2.** Local structure parameters around Co in samples calculated from the EAXFS data.

**Table S3.** Production rate of catalysts normalized by surface area.

**Table S4.** Gibbs free energy of reduced species during CO_2_ reduction.

**Table S5.** Reaction energies ΔG (eV)) of each elementary step involved in the CO_2_PR on the CoIn-LDH/MOF, CoIn-LDH and CoIn-LDH-3.

**Table S6.** Bader charge analysis of metal atoms in the CoIn-LDH/MOF.

**Table S7.** Bader charge analysis of metal atoms in the CoIn-LDH.

**Table S8.** Bader charge analysis of metal atoms in the CoIn-LDH-3.

**Experimental Section**

**Materials:** In(NO_3_)_3_∙4.5H_2_O, triethanolamine (TEOA), hexamethylenetetramine (C_6_H_12_N_4_), NaSO_4_ were purchased from Sinopharm Chemical Reagent. In(NO_3_)_3_∙ 6H_2_O was purchased from Beijing Warwick Chemical Co., Ltd. (CH_3_COO)_2_Co∙2H_2_O, Co(NO_3_)_3_∙6H_2_O were purchased from Shanghai Macklin Biochemical Technology Co., Ltd. N,N-dimethylformamide (DMF), absolute ethanol (C_2_H_5_OH) and acetonitrile (CH_3_CN) were purchased from Tianjin Damao Chemical Reagent Factory. 2-Aminoterephthalic acid (H_2_BDC-NH_2_, 99.87%) was purchased from **Bide Pharmatech Ltd.** 2-Aminoterephthalic acid (H_2_BDC-NH_2_, 98%), Ru(bpy)_3_Cl_2_∙6H_2_O (bpy=2’2-bipyridine) were purchased from Shanghai Aladdin Biochemical Technology Co., Ltd. Ammonium fluoride (NH_4_F), urea (CO(NH_2_)_2_) were purchased from Energy Chemical. All materials and reagents were used directly without any further purification. Deionized water was used in all the experimental processes.

**Synthesis of MIL-68(In)-NH_2_:** 0.2979 g of In(NO_3_)_3_∙4.5H_2_O, 0.585 g of 2-aminoterephthalic acid (98%, H_2_BDC-NH_2_) and 0.585 g of 2-aminoterephthalic acid (99.87%, H_2_BDC-NH_2_) were together added into 20 mL of DMF and stirred vigorously for 1.6 h at room temperature. Then the mixed solution was transferred to 50 mL Teflon-lined autoclave at 125 ℃ for 5 h. After cooled down to room temperature, the yellow solid products were filtered and washed with absolute ethanol. Finally, the precipitates were dried at 60 ℃ overnight.

**Synthesis of CoIn-LDH/MOF:** First, 0.448 g of (CH_3_COO)_2_Co∙2H_2_O, 0.02g of CO(NH_2_)_2_, 0.0041 g of NH_4_F, 15 mL of C_2_H_5_OH and 5 mL of deionized water were mixed and stirred well. Then, the obtained MIL-68(In)-NH_2_ was dispersed in the above solution with ultrasound treatment for 30 min. Finally, the mixture was transferred to 50 mL Teflon-lined autoclave and maintained at 100 °C for 6 h, and the products were filtered, washed thoroughly with deionized water, absolute ethanol and dried at 60 °C overnight.

**Synthesis of CoIn-LDH:** 0.51g of In(NO_3_)_3_∙6H_2_O, 1.09 g of Co(NO_3_)_3_∙6H_2_O were dissolved in 20 mL of deionized water, and added hexamethylenetetramine. After being stirred for 15 min to make it well-mixed, then the mixture was transferred to 50 ml Teflon-lined autoclave, heated at 100 ℃ for 8 h. Finally, the obtained product with absolute ethanol and deionized water were washed for three times each and dried at 60 °C overnight.

**Characterization:** Powder X-ray diffraction (PXRD) patterns of the samples were collected on a Rigaku MinFlex600 diffractometer with a Cu Kα radiation (*λ* = 1.5405Å). Hitachi Regulus 8100 field emission scanning electron microscope (SEM) and FEI talos f200x transmission electron microscopy (TEM) was used to analyze the morphologies of different samples. X-ray photoelectron spectroscopy (XPS) was performed on the AXIS-SUPRA+ spectrometer with a monochromatic Al Kα source. The binding energies were determined by the C 1s spectrum as a reference at 284.8 eV. ICP-OES analysis was got on a PlasmaQuant 9100. The photoluminescence (PL) spectra were acquired using a Hitachi Analytical Instrument (F-7000) at room temperature. UV-vis diffuse reflectance (UV-vis DRS) were conducted on a UV-vis spectrophotometer (PerkinElmer UV/VIS/NIR Spectrometer Lambda 1050+) with a wavelength range of 250–800 nm via an integrator sphere by using BaSO_4_ as a reference. Electron paramagnetic resonance (EPR) spectroscopic measurement was performed at room temperature using Bruker A300.

*In situ* infrared Fourier transform spectroscopy measurements (*In situ* DRIFT) were performed on a Perkin Elmer Spectrum 3. First, the catalyst was pretreated at 120 °C for 1 h in the Ar atmosphere to remove the surface pollutant. Then CO_2_ (40 mL/min) with bubbled H_2_O was introduced into the chamber at 20 ℃ for 30 min, and the background spectra were recorded. Finally, spectra were recorded under light.

*In situ* X-ray photoelectron spectroscopic (XPS) spectra were obtained from an electron spectrometer (ESCALAB 250X) under the light irradiation (UVEC-4Ⅱ).

**Photocatalytic CO_2_ reduction**: The CO_2_PR were performed in a closed quartz glass reactor (volume: 150 mL) with a window on the top for visible-light irradiation. The whole system was composed of [Ru(bpy)_3_]Cl_2_·6H_2_O (3.3 mg), samples (10 mg) and solvent [30 mL in total, CH_3_CN/TEOA/H_2_O = 3:1:1 (volume ratio)]. First, open up the gas inlet/outlet valve, and CO_2_ was filled into reactor by bubbling. Then the temperature of the reactor was maintained at room temperature by thermostatic water circulation, and the performance of all catalysts was tested under irradiation of 300 W Xenon lamp (λ>400 nm). Finaly, the gaseous products were detected by PannaA91-Plus chromatograph equipped with TCD and FID detectors. The isotopic experiment was carried out under the same condition except using ^13^CO_2_ and gas chromatography-mass spectrometry (8890-5977B GC-MS instrument, Agilent Technologies, USA), to check the product.

**Electrochemical test:** A three-electrode system was used to conduct electrochemical experiments on the electrochemical workstation (CHI760A), platinum-plate electrode and saturated calomel electrode (SCE) were used as the counter electrode and reference electrode, respectively. The working electrode was obtained by coating the photocatalyst dispersed in the solution (photocatalyst: 6 mg, H_2_O: 675 μL, C_2_H_5_OH: 275 μL, Nafion 50 μL) on the FTO glass. The electrodes were measured using 0.1 M Na_2_SO_4_ as aqueous solution.

**Density functional theory (DFT) calculations:** All functional theory (DFT) method were performed using the Vienna *ab initio* simulation package (VASP) code with the projector augmented wave (PAW) method.^[1, 2]^ The generalized gradient approximation (GGA) combined with Perdew-Burke-Ernzerhof (PBE) functional was employed to describe the exchange-correlation term.^[3]^ The projector augmented wave (PAW) pseudo-potentials were used to describe ionic cores.^[2]^ The cutoff energy for the plane-wave basis was set to 450 eV. The Van der Waals (vdW) interactions was described by using the empirical correction in Grimme’s scheme (DFT-D3) in all calculations.^[4]^ The convergence tolerances for energy and force were set to 10^−5^ eV and 0.05 eV/Å, respectively. The Gibbs free energy change (ΔG) for each elemental step was defined as

ΔG = ΔE_DFT_ + ΔE_ZPE_ - TΔS

In this equation, ΔE_DFT_ is denotes the electronic energy change directly obtained from DFT calculations, ΔE_ZPE_ and ΔS are the zero-point energy correction and entropy change obtained from frequency calculations at 298.15 K.


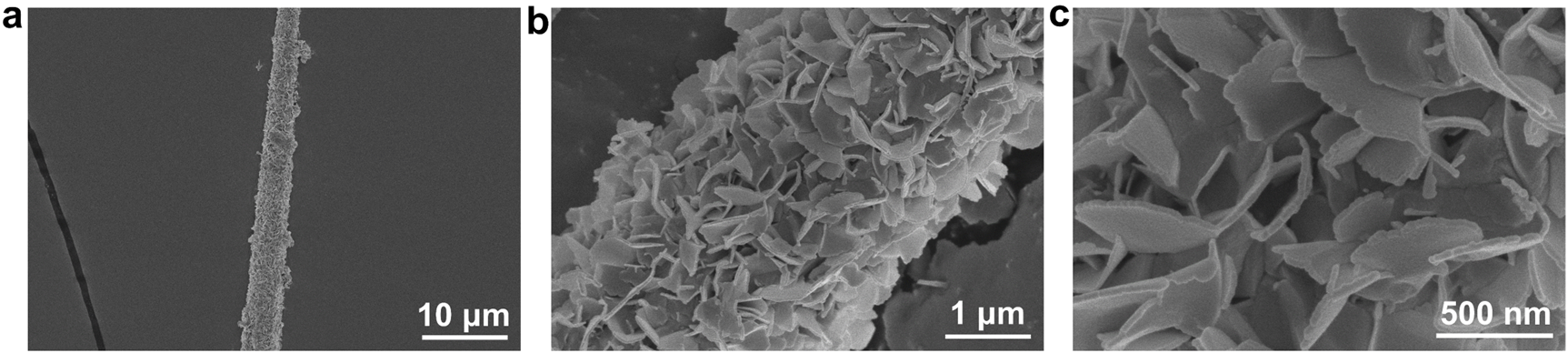


**Figure S1.** SEM images of CoIn-LDH/MOF.


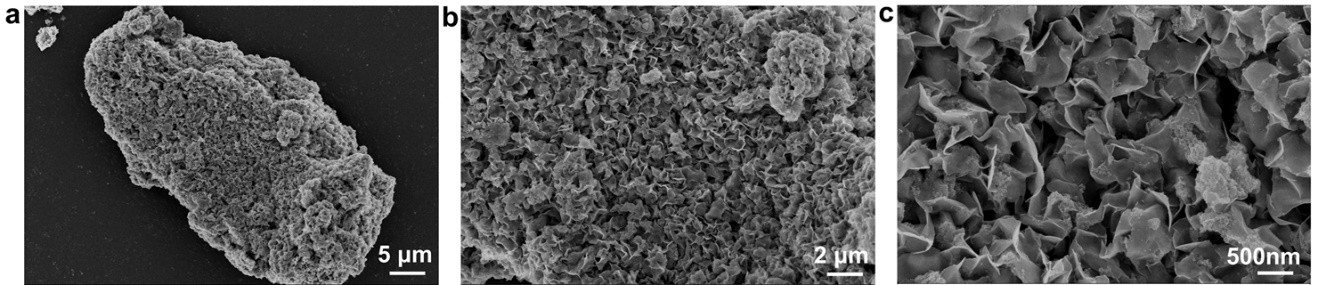


**Figure S2.** SEM images of CoIn-LDH.


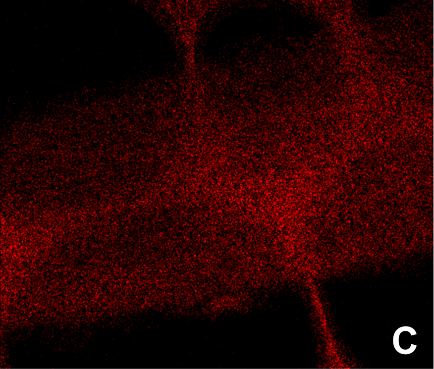


**Figure S3.** Elemental mapping image of CoIn-LDH/MOF.


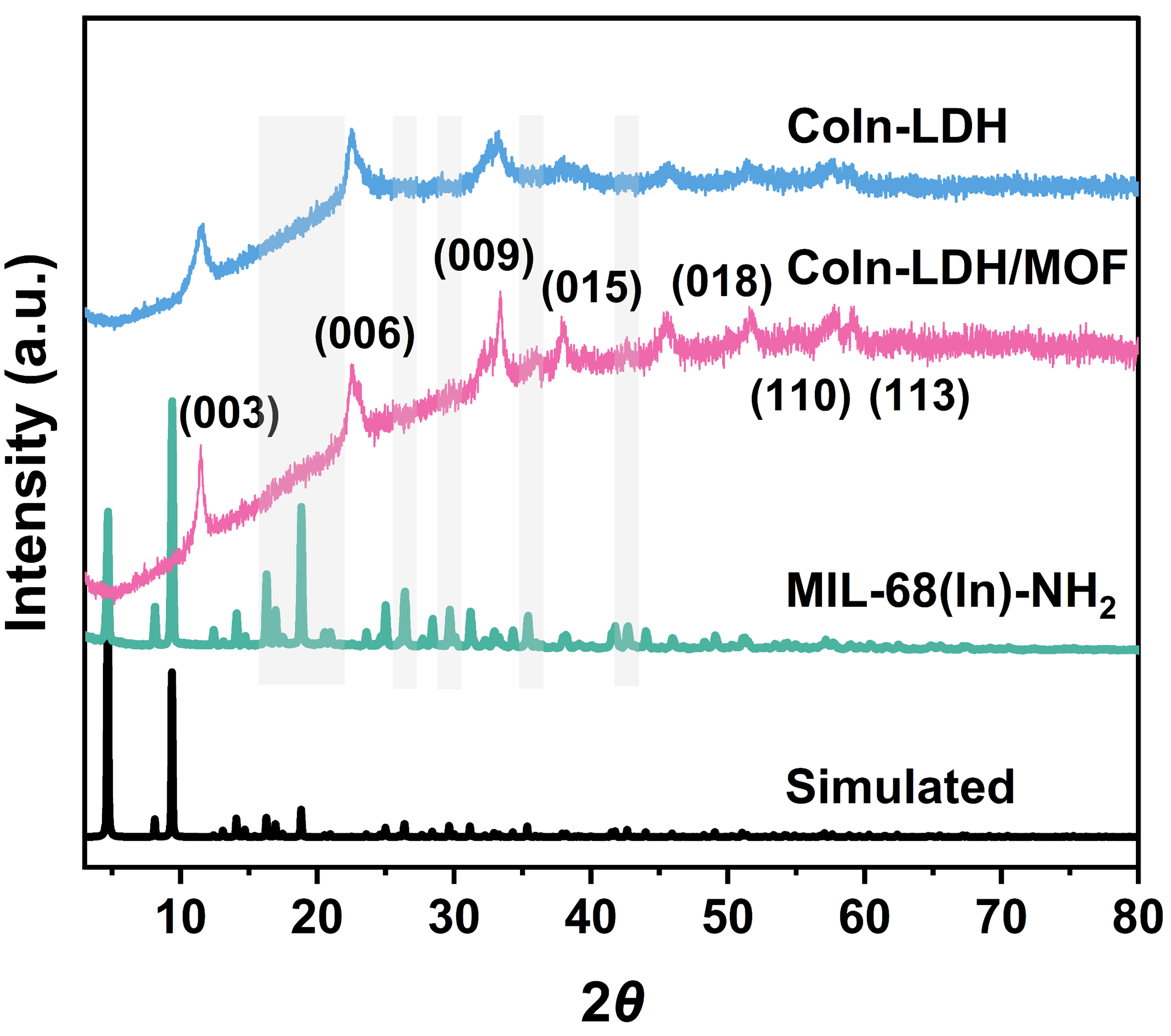


**Figure S4.** PXRD patterns for MIL-68(In)-NH_2_, CoIn-LDH and CoIn-LDH/MOF.


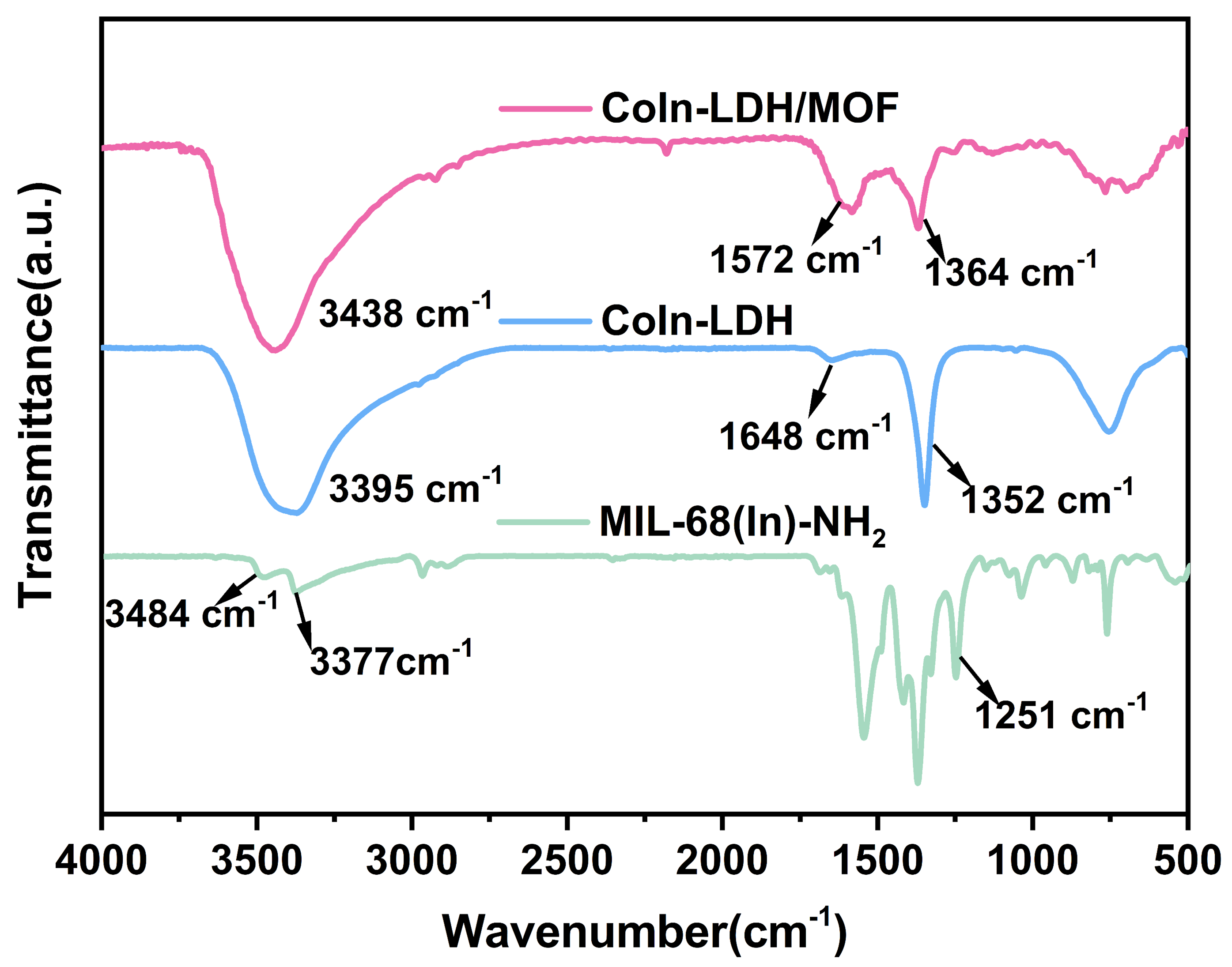


**Figure S5.** FT-IR spectra of MIL-68(In)-NH_2_, CoIn-LDH and CoIn-LDH/MOF.


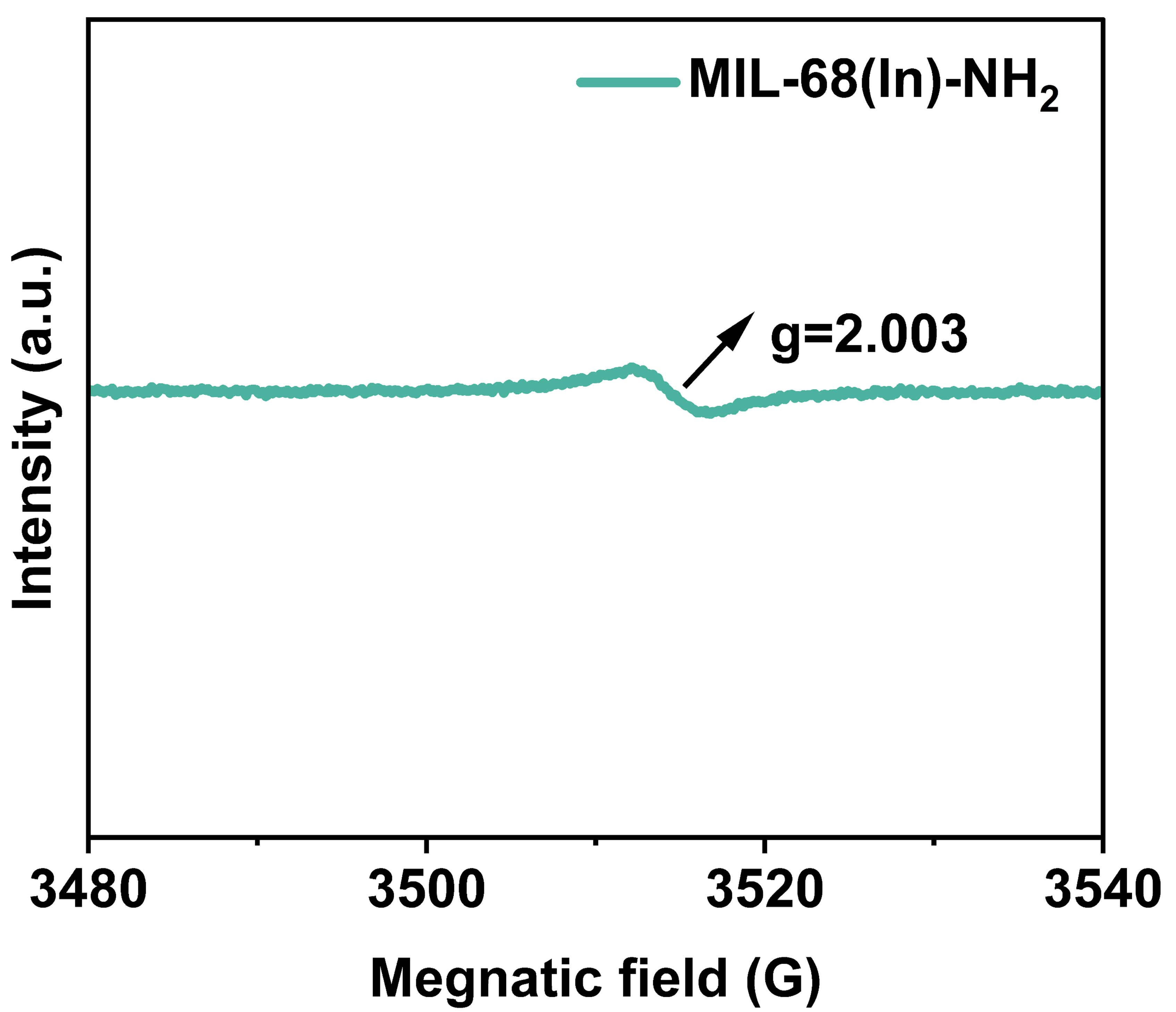


**Figure S6.** EPR spectra of MIL-68(In)-NH_2_.


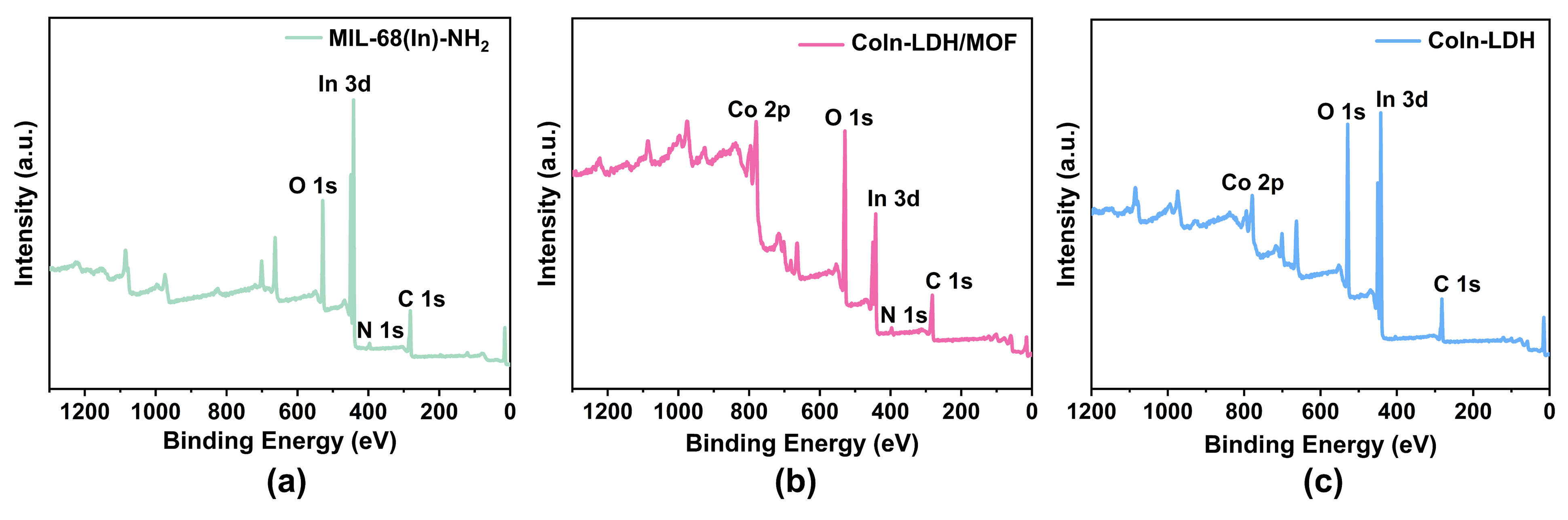


**Figure S7.** XPS survey spectra of (a)MIL-68(In)-NH_2_, (b)CoIn-LDH/MOF, (c) CoIn-LDH.


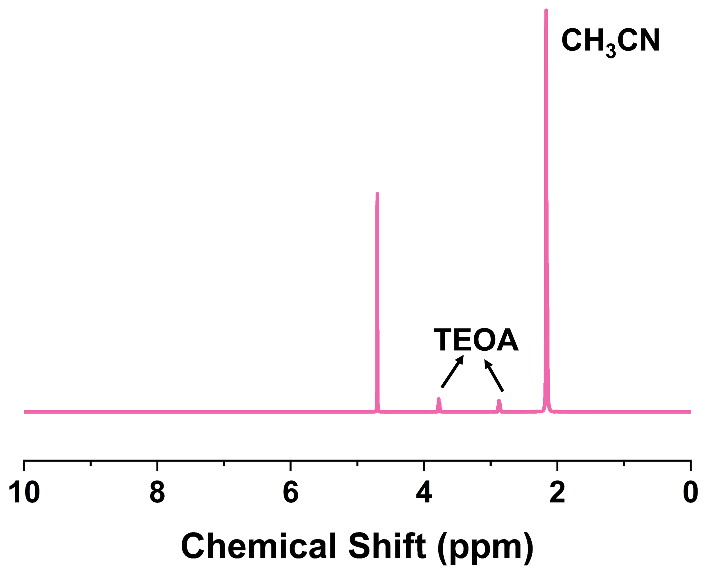


**Figure S8.** ^1^H NMR spectra of the reaction system following CO_2_ reduction under illumination (*λ* > 400 nm).


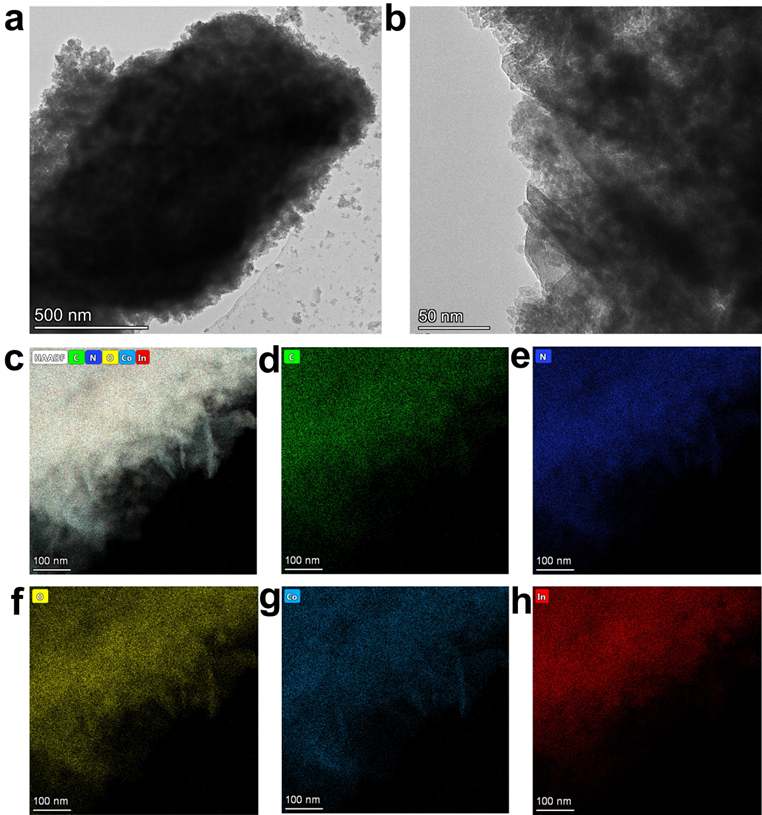


**Figure S9. (**a-b**)** TEM images, (c-h) elemental mapping of CoIn-LDH/MOF after CO_2_ reduction reaction.


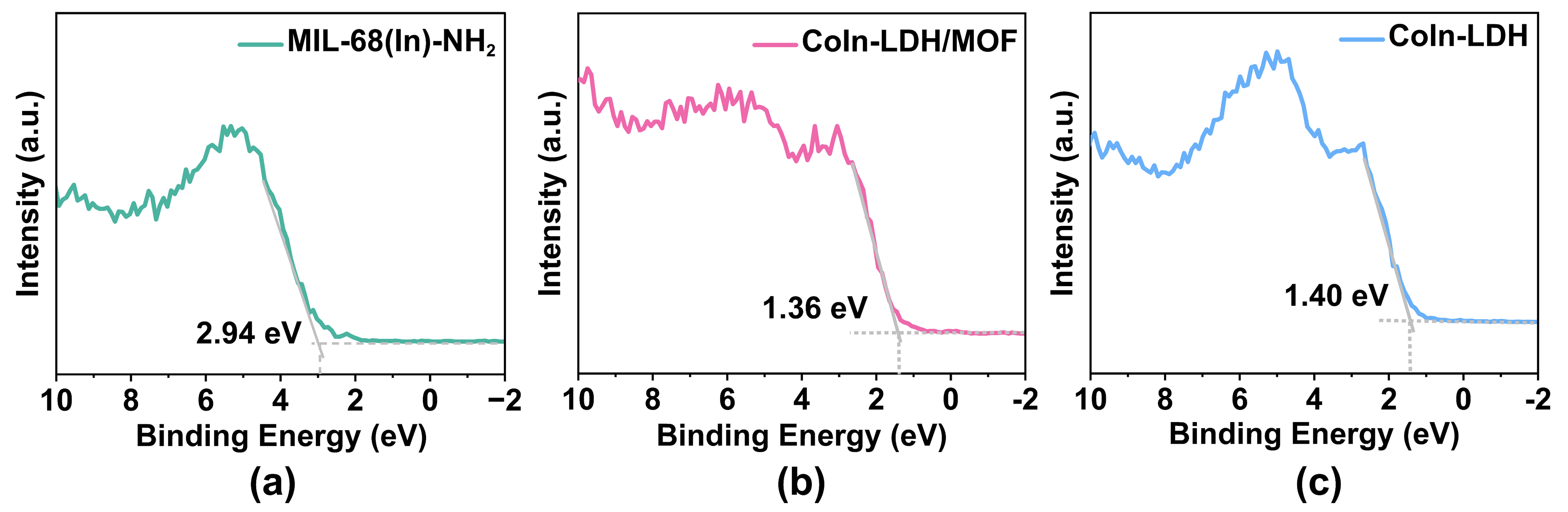


**Figure S10.** Valence band XPS spectra of (a) MIL-68(In)-NH_2_, (b) CoIn-LDH/MOF, (c) CoIn-LDH.


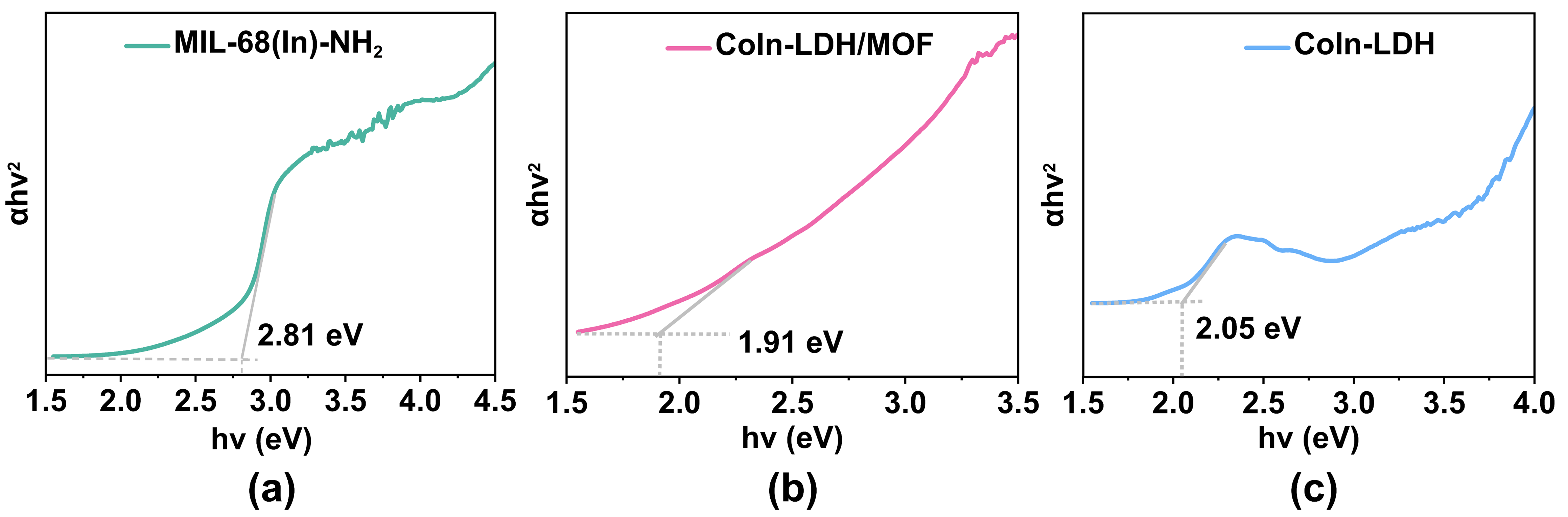


**Figure S11.** Tauc plots of (a) MIL-68(In)-NH_2_, (b) CoIn-LDH/MOF, (c) CoIn-LDH.


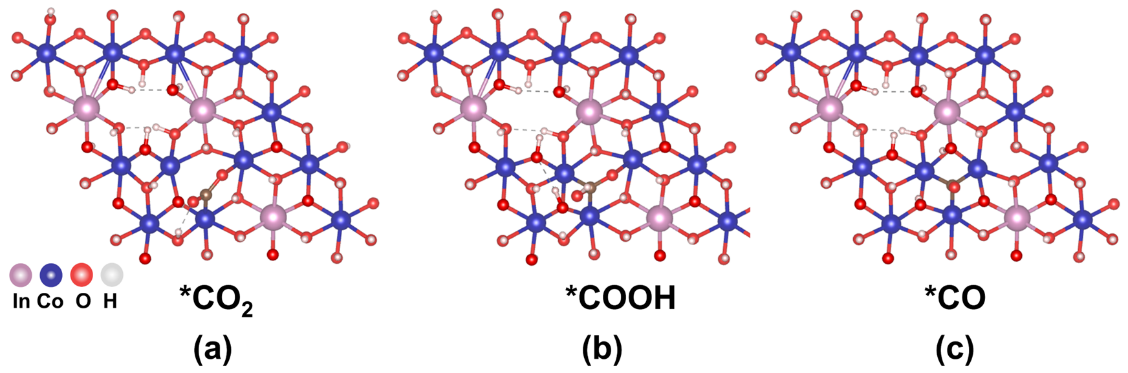


**Figure S12.** Optimized geometries of photocatalytic CO_2_ reduction intermediates for CoIn-LDH/MOF.


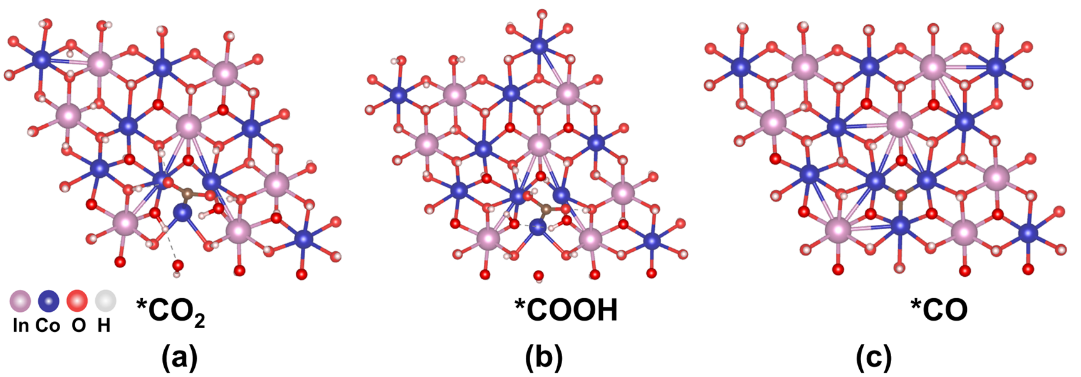


**Figure S13.** Optimized geometries of photocatalytic CO_2_ reduction intermediates for CoIn-LDH.


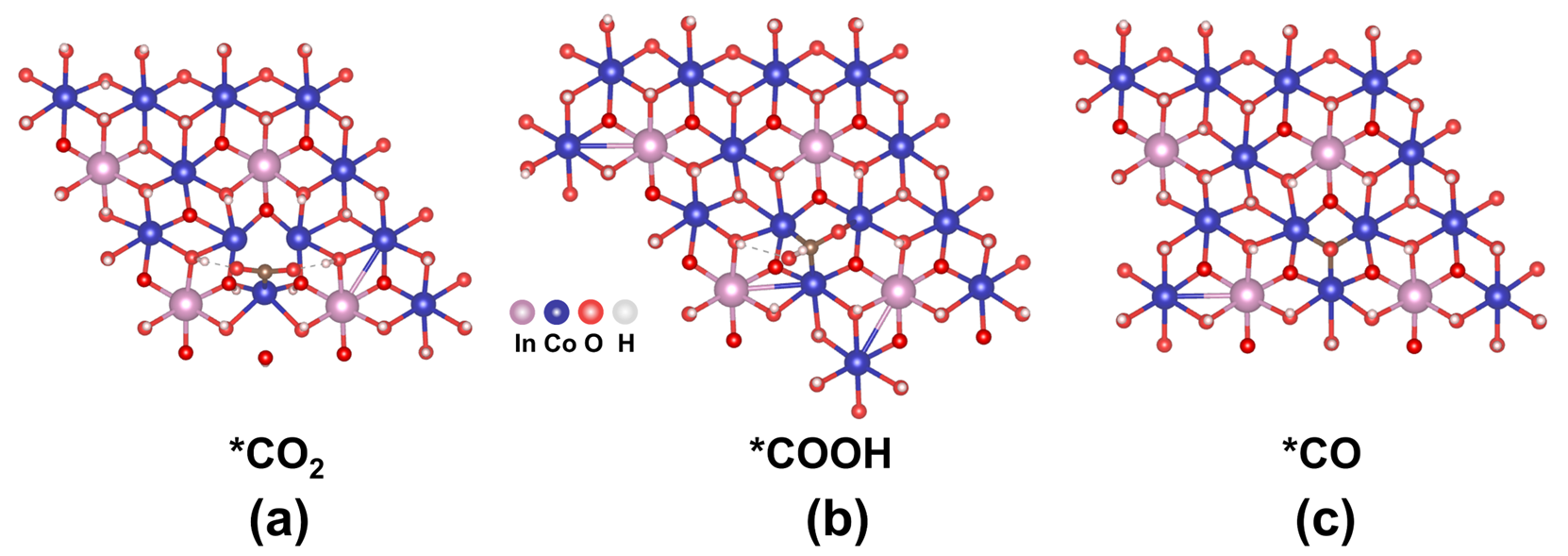


**Figure S14.** Optimized geometries of photocatalytic CO_2_ reduction intermediates for CoIn-LDH-3.

**
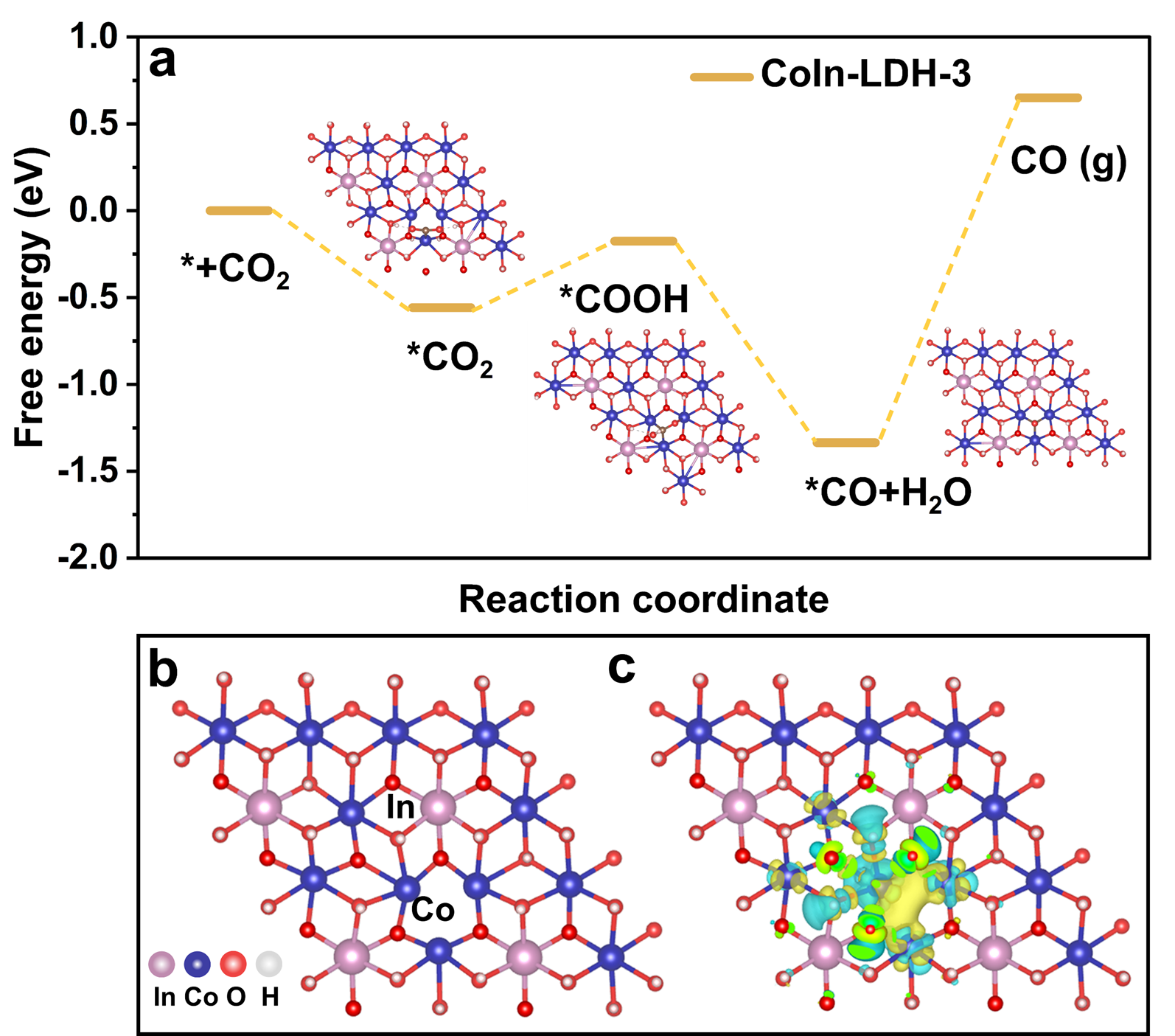
**

**Figure S15.** (a) Gibbs free energy diagram showing the photocatalytic reduction of CO_2_ to CO using CoIn-LDH-3. (b) Bader charge analysis, (c) Differential charge density maps of CoIn-LDH-3 (isosurface level: 0.0025 eV/Å^3^) (yellow and blue regions indicate electron accumulation and depletion, respectively).


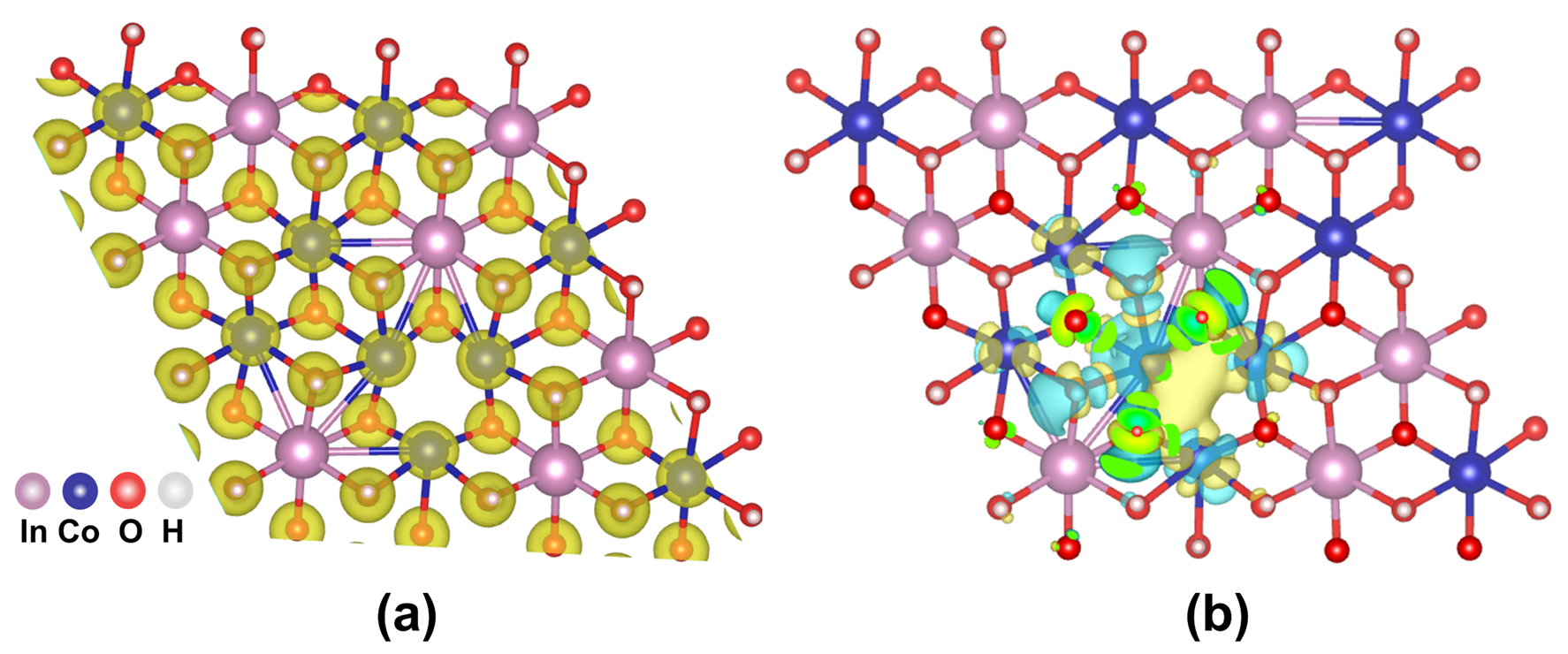


**Figure S16.** (a) Charge density distribution of CoIn-LDH (isosurface level: 0.192 eV/Å^3^). (b) Differential charge density maps of CoIn-LDH (isosurface level: 0.0025 eV/Å^3^) (yellow and blue regions indicate electron accumulation and depletion respectively).


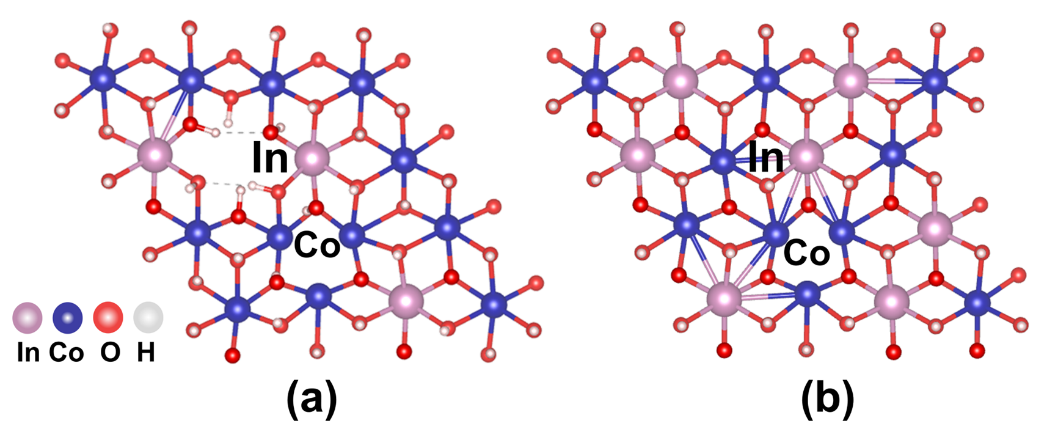


**Figure S17.** Bader charge analysis of (a) CoIn-LDH/MOF, (b) CoIn-LDH.

**Table S1.** Summary of elemental analyses data for CoIn-LDH/MOF and CoIn-LDH from ICP results.

| Sample | CoIn-LDH/MOF | CoIn-LDH |
| --- | --- | --- |
| Co/In | 2.6 | 1.2 |

**Table S2.** Local structure parameters around Co in samples calculated from the EAXFS data.

| Sample | Shell | Coordination number | R (Å) | Debye-Waller facto σ^2^ (Å^2^) | Amplitude attenuation factor S_0_^2^ |
| --- | --- | --- | --- | --- | --- |
| Co foil | Co-Co | 12 | 2.49±0.01 | 0.0059±0.0002 | 0.702 |
| Co_3_O_4_ | Co-O | 4.0±0.2 | 1.92±0.01 | 0.0021±0.0006 | 0.702 |
|  | Co-Co | 3.8±0.4 | 2.85±0.01 | 0.0037±0.0007 |  |
|  | Co-Co | 8.1±0.9 | 3.36±0.01 | 0.0070±0.0009 |  |
| CoIn-LDH/MOF | Co-O | 3.7±0.4 | 1.97±0.01 | 0.0122±0.0016 | 0.702 |
|  | Co-M | 4.6±1.0 | 2.95±0.01 | 0.0237±0.0040 |  |
| CoIn-LDH | Co-O | 6.4±0.6 | 2.02±0.01 | 0.0154±0.0015 | 0.702 |
|  | Co-M | 5.7±0.7 | 3.14±0.01 | 0.0141±0.0014 |  |

**Table S3.** Production rate of catalysts normalized by surface area.

| Productivity(mmol m^-2^ h^-1^) | CO | H_2_ |
| --- | --- | --- |
| CoIn-LDH/MOF | 0.0227 | 0.0063 |
| CoIn-LDH | 0.0239 | 0.014 |

**Table S4.** Gibbs free energy of reduced species during CO_2_ reduction.

| Sample | G(eV) | | | |
| --- | --- | --- | --- | --- |
|  | *CO_2_ | *COOH | *CO | CO |
| CoIn-LDH/MOF | -0.55 | -0.11 | -0.97 | 0.65 |
| CoIn-LDH | -1.63 | -0.98 | -1.18 | 0.65 |
| CoIn-LDH-3 | -0.56 | -0.16 | -1.33 | 0.65 |

**Table S5.** Reaction energies ΔG (eV)) of each elementary step involved in the CO_2_PR on the CoIn-LDH/MOF, CoIn-LDH and CoIn-LDH-3.

| Reactions △G(eV) | CoIn-LDH/MOF | CoIn-LDH | CoIn-LDH-3 |
| --- | --- | --- | --- |
| CO_2_+*+2H^+^+2e^-^→*CO_2_ | -0.55 | -1.63 | -0.56 |
| *CO_2_+2H^+^+2e^-^→*COOH+ H^+^+e^-^ | 0.44 | 0.65 | 0.38 |
| *COOH + H^+^ + e^-^→*CO + H_2_O | -0.86 | -0.2 | -1.17 |
| *CO → CO + * | 1.62 | 1.83 | 1.98 |

**Table S6.** Bader charge analysis of metal atoms in the CoIn-LDH/MOF.

| Element | ZVAL | Charge | Valance |
| --- | --- | --- | --- |
| Co | 9 | 8.0725 | 0.9275 |
| In | 3 | 1.3454 | 1.6546 |

^a)^ ZVAL: ideal number of valence electrons; ^b)^ Charge: the number of valence electrons actually calculated;

^c)^ Valance: ZVAL subtracting Charge.

**Table S7.** Bader charge analysis of metal atoms in the CoIn-LDH.

| Element | ZVAL | Charge | Valance |
| --- | --- | --- | --- |
| Co | 9 | 8.2207 | 0.7793 |
| In | 3 | 1.3751 | 1.6249 |

^a)^ ZVAL: ideal number of valence electrons; ^b)^ Charge: the number of valence electrons actually calculated;

^c)^ Valance: ZVAL subtracting Charge.

**Table S8.** Bader charge analysis of metal atoms in the CoIn-LDH-3.

| Element | ZVAL | Charge | Valance |
| --- | --- | --- | --- |
| Co | 9 | 8.2121 | 0.7879 |
| In | 3 | 1.3529 | 1.6471 |

^a)^ ZVAL: ideal number of valence electrons; ^b)^ Charge: the number of valence electrons actually calculated;

^c)^ Valance: ZVAL subtracting Charge.

Supporting References

[1] G. Kresse, J. Furthmüller, *Phys. rev. B* **1996**, *54*, 11169.

[2] G. Kresse, D. Joubert, *Phys. Rev. B* **1999**, *59*, 1758.

[3] J. P. Perdew, K. Burke, M. Ernzerhof, *Phys. Rev. Lett.* **1996**, *77*, 3865.

[4] S. Grimme, J. Antony, S. Ehrlich, H. Krieg, *J. Chem. Phys.* **2010**, *132*, 154104.
